# The Optimal Route of Administration of the Glycoprotein IIb/IIIa Receptor Antagonist Abciximab During Percutaneous Coronary Intervention; Intravenous Versus Intracoronary

**DOI:** 10.2174/157340308786349480

**Published:** 2008-11

**Authors:** Allan Iversen, Søren Galatius, Jan S Jensen

**Affiliations:** Department of Cardiology, Gentofte University Hospital, Copenhagen, Denmark

**Keywords:** Abciximab, coronary heart disease, glycoprotein IIb/IIIa, intracoronary, intravenous, percutaneous coronary intervention.

## Abstract

The use of the glycoprotein (GP) IIb/IIIa receptor antagonist Abciximab has over the years become an important part of the anticoagulant regimen in patients with acute coronary syndrome undergoing percutaneous coronary intervention. Abciximab is a potent inhibitor of platelet aggregation and thrombus formation, but other mechanisms, such as suppression of the inflammatory pathways, have also been proposed to contribute to the benefits of Abciximab.

The optimal route of administration, i.e. intravenous versus intracoronary, of the first dose has been questioned, but only tested in small, non-randomised and retrospective studies or studies with short follow-up. No definite conclusion can be made based on these studies.

In this review we present the current knowledge published about the intracoronary administration of Abciximab including the mechanisms behind the potential beneficial effects, and the safety. The emphasis will be on clinical trials rather than on studies on the pharmacological mechanisms, as the latter have been reviewed thoroughly elsewhere.

Our conclusion from this present review is that randomized trials of intracoronary versus intravenous bolus of Abciximab are needed.

## INTRODUCTION

Cardiovascular diseases are responsible not only for the majority of deaths in the United States [1] and Europe, but also for a large proportion of the hospital expenses. Atherosclerosis in the coronary arteries leading to acute coronary syndrome (ACS) accounts for the majority of cardiovascular diseases and massive resources have been put into the management of ACS. Along with improvement of mechanical devices such as stents, focus is directed to periprocedural anticoagulants, so-called facilitated percutaneous coronary intervention (PCI). One class of drug in facilitated PCI is the glycoprotein (GP) IIb/IIIa receptor antagonist of which Abciximab is one of them. Abciximab was released for commercial use in February 1995.

Numerous interventional trials have proven Abciximab effective in reducing death, myocardial infarction (MI) and revascularization in patients with ACS. These trials, the first of them published in the 1990ies, and the latest ISAAR REACT 2 which was published in 2008 [2], are based on the use of intravenous (iv) Abciximab, exclusively. However, during the recent years, both a number of case-reports and a few smaller non randomised studies have been published, suggesting that intracoronary (ic) administration of Abciximab may be a better alternative.

The main purpose of this article is to review published clinical studies presenting data on the use of intracoronary administration of Abciximab.

## BACKGROUND - CLINICAL STUDIES ON ABCIXIMAB

It has been known for years that activation followed by aggregation of platelets to a thrombus is the major pathophysiological player causing ACS [3,4]. The first event in a cascade of events leading to ACS is the rupture of an atherosclerotic plaque in a coronary artery, thereby uncovering subendothelial connective tissue in the arterial wall to the components of the blood, especially platelets. On the platelet surface the glycoprotein IIb/IIIa receptor is expressed, which is one of the surface proteins responsible for platelet-aggregation. Abciximab irreversibly inhibits this aggregation.

Previous studies have proven the beneficial effect of Abciximab in ACS and PCI. The first large-scaled, multicenter, randomized, double-blind trial was the Evaluation of 7E3 for the Prevention of Ischemic Complication study (EPIC) published in 1994 [5]. In this study 2,100 high-risk patients (unstable angina pectoris (UAP), ST-elevation MI (STEMI) or high-risk coronary lesions) undergoing PCI received iv bolus of Abciximab or placebo followed by a 12-hour iv-infusion of either Abciximab or placebo. The results were convincing in advantage of Abciximab with respect to the composite end-point consisting of death, MI or revascularization demonstrating a significant reduction from 12.8% to 8.3% after 30 days. Even after 6 months and 3 years there was a significant reduction in the composite end-point among those treated with Abciximab from 35.1% to 27.0% [6] and 47.2% to 41.1% [7], respectively. In the 30-day period a doubling of major bleeding was observed in the active drug group from 6.6% to 14.0% [8] - one major problem in adding an extra anticoagulant to the angioplasty-regimen.

The EPIC trial was followed by Evaluation in Percutaneous Transluminal Coronary Angioplasty to Improve Long-Term Outcome with Abciximab GP IIb/IIIa Blockage – EPILOG [9] and Evaluation of Platelet IIb/IIIa Inhibitor for Stenting – EPISTENT [10,11]. In EPILOG the same composite end-point as in EPIC was used, but the enrolement criteria were expanded to include low-risk patients as well as high-risk patients. Not only did the investigators randomize patients to either Abciximab or placebo. An additional regimen of low-dose heparin (70 U/kg; activated clotting time (ACT)≥200 sec) versus standard heparin (100 U/kg; ACT≥300 sec) was tested. Since the EPIC trial convincingly proved the effect of Abciximab in patients with UAP, STEMI or planned PCI, those patients were excluded from the EPILOG trial. 4,800 patients were planned to be enrolled, but the study was terminated prematurely (with nearly 2,800 patients enrolled) because an interim analysis at 30 days showed a 56% reduction with respect to the composite end-point among those receiving Abciximab (Abciximab iv-bolus followed by 12 hours of iv-infusion regardless of standard- or low-dose heparin) compared to those receiving placebo. The beneficial outcome in this treatment group persisted after 6 months follow-up [12]. No differences were found in bleeding complications. The results were used to extrapolate the results from EPIC to low-risk patients undergoing PCI. In the EPISTENT trial 2,400 patients were randomized to stenting plus Abciximab, stenting plus placebo or balloon-angioplasty plus Abciximab. Follow-up was at 30 days and a composite end-point of death, MI or need for urgent revascularization was used. The study showed a reduction in the composite end-point from 10.8% in the stent plus placebo to 5.3% in the stent plus Abciximab. In the balloon plus Abciximab group 6.9% reached the composite end-point. Abciximab was found to be safe with respect to major bleeding.

Cho *et al*. made a pooled analysis of the three studies mentioned above [13] to demonstrate whether Abciximab had the same beneficial effect in both men and women. No differences were found. As part of their analysis a multivariate logistic regression was performed. Gender was not found to be associated to either increased or reduced risk of the composite end-point. Furthermore, it was found in this pooled analysis (n=6,995) that the use of Abciximab was associated with a 49% relative risk reduction of reaching the end-point.

In C7E3 Fab Antiplatelet Therapy in Unstable Angina - CAPTURE, another interventional study, the question of whether Abciximab should be started up front and prior to PCI, was investigated [14]. In this study, Abciximab or placebo was started as iv-infusion 18-24 hours prior to PCI in contrast to earlier regimes in which Abciximab was given as an iv-bolus, at the time of PCI. Because of a 29% reduction of the composite end-point at 30 days in the Abciximab arm, the study was terminated prematurely, when 1,265 (of expected 1,400) patients had been enrolled. However, at 6 months no differences between Abciximab and placebo were observed. Based on this, pre-PCI Abciximab-infusion is at generally not recommended. Most trials on GP IIb/IIIa inhibitors have been conducted on patients scheduled for PCI. In 2002 Boersman *et al*. published a meta-analysis of trials with patients receiving GP IIb/IIIa inhibitors and not routinely scheduled for PCI [15]. The main findings of this large analysis (n=31,402) was a 9 % reduction in death and MI in patients receiving GP IIb/IIIa inhibitors.

There have been some safety concerns about the use of Abciximab. Especially with respect to both acute [16,17] and delayed [18] thrombocytopenia leading to excess risk of bleeding. Baseline characteristics such as low body weight, high age and low baseline platelet counts increase the risk of thrombocytopenia [19].

Since Abciximab is a human chimeric Fab fragment antibody the question of development of hypersensitivity has been investigated. Tcheng *et al*. [20] found that in patients receiving a second dose of Abciximab, the number of human antichimeric antibody-positive rose from 4.8 % before first administration to 19.0 % after second administration. However, this had no influence on clinical outcome. For the same reason Madan *et al*. [21] investigated if the efficacy would decline when Abciximab was given more than once, and found no such relationship.

In general Abciximab is thought to be safe with respect to minor and major bleedings, despite an increase in the incidence of bleedings in some studies [8,22-26]. The same is observed for the risk of stroke [27], both compared to placebo and to another GP IIb/IIIa inhibitor, Eptifibatide [28].

Oral GP IIb/IIIa inhibitors have been considered and tested (EXCITE [29], OPUS-TIMI 16 [30], SYMPHONY [31], 2^nd^ SYMPHONY [32] and BRAVO [33]), all with adverse outcomes [31,34,35]. In a meta-analysis including the above mentioned trials Chew *et al*. [35] found a 37 % increase in mortality in oral GP IIb/IIIa inhibitors compared to placebo. The results were surprising, since one would expect a similar positive effect as seen in iv administration. In 2004 Sy *et al*. [36] presented several hypothesis regarding these finding, but no trials on oral GP IIb/IIIa inhibitors have been published since.

An important issue, which must be taken into consideration when discussing the use of GP IIb/IIIa inhibitors, is the use of other anticoagulants, such as thienopyridines and newer agents as Prasugrel and Cangrelor. In 2008 the ISAR-REACT 2 trial 1 year follow up was published [2]. In this trial all patients (n=2,022) with non-ST-segment elevation acute coronary syndromes received 600 mg clopidogrel pretreatment before PCI. Approximately one third received Abciximab iv in relation to PCI. After one year the primary end point of death, MI, or target vessel revascularization (TVR) was reached in 23.3 % of patients allocated to Abciximab *vs*. 28.0 % of patients allocated to placebo (P=0.0012). The were no safety issues [37]. Regarding newer agents one must consider their potential interaction with GP IIb/IIIa inhibitors also in the context of iv *vs*. ic administration.

In the latest European guidelines for treatment of ACS the use of GP IIb/IIIa inhibitors are described, but only trials concerning the iv route have been taken into consideration in the ECS recommendations [38]. This is also the case for the American Heart Association guidelines [39,40].

The pharmacological mechanism of Abciximab has recently been described [41] and are summarised in Table **1** below.

Most important in this setting probably is the fact that high local concentration of Abciximab seems to have a thrombolytic effect [42]. Marciniak *et al*. have shown that low concentrations of Abciximab [1.3-3 µg/ml] prevents further platelet aggregation in a thrombus, while high concentrations (≥ 10 μg/ml) have the ability to disperse an already formed thrombus.

Despite this iv administration of Abciximab is generally recommended, although there may be arguments for ic bolus administration. We therefore reviewed clinical studies and case reports on this issue. 

## METHODS

A search in PubMed and EMBASE was performed using the search criteria Abciximab, Reo-Pro, glycoprotein IIb/IIIa, intracoronary, acute coronary syndrome, percutaneous coronary intervention and coronary angioplasty. In an effort to identify further published trials we searched reference lists and to identify ongoing trials we searched in www.clinical trials.gov, and Cochrane Central Register of Controlled Trials (CENTRAL) was consulted for reviews. The results were narrowed down to include case reports and clinical trials testing or describing the issue of intracoronary administration of Abciximab.

## TRIALS EVALUATING INTRAVENOUS *VS*. INTRACORONARY ABCIXIMAB

The first reports on ic administered Abciximab were presented in 1997. Bailey *et al*. described a small study of 12 patients with ´unstable clinical syndromes´ (defined as: subacute stent thombosis, UAP or Post-MI) who received ic delivered Abciximab [43]. Inspired by studies in which urokinase [44] and heparin [45] was given ic with positive results on outcome, Bailey treated 12 patients with angioscopic verified thrombus with 5-10 mg ic delivered Abciximab in addition to orally given aspirin and iv administered heparin. The ic administered Abciximab was not followed by iv infusion. In 11 of 12 patients resolution of the thrombus was observed after performing re-angioscopy. The patients were then treated with mechanical revascularization. The number of patients was too small to evaluate major complications such as STEMI, Non-STEMI, death and emergency coronary artery by-pass grafting (CABG). In 1999 Bartorelli *et al*. reported a case [46] in which Abciximab was administered ic in a STEMI-patient with occlusion of proximal LAD. Treatment with systemic thrombolysis and angioplasty was attempted without success prior to the ic delivery of Abciximab. After ic administration of the GP IIb/IIIa blocker complete resolution of the thrombus was seen with subsequent TIMI 3 flow. No bleeding complications were described. Through 1999 to 2004 several cases and small studies on the matter were published.

In 2000 Baron *et al*. investigated the possibility of coating coronary stents with Abciximab (c7E3-Fab) and found that the drug eluted slowly and in a predictable manner and significantly inhibited platelet deposition *in vitro* [47]. Abciximab-eluting stents for commercial use is to our knowledge not available. Barsness *et al*. found that in 57 patients with >60% stenosis in a saphenous vein graft that required percutaneous intervention, local delivery of Abciximab resulted in significantly reduced thrombus size evaluated on angiogram. One third of the patients had no visible thrombus at the initiation of the procedure, while two thirds showed no signs of thrombus after local delivery of Abciximab. At the end of the procedure 89% of the patients were without thrombus [48].

In 2003 at the convention of Transcatheter Cardiovascular Therapeutics - TCT, Kennon *et al*. presented in abstract form a case series of 6 patients with no-reflow after PCI treated with local delivery of Abciximab. All patients showed improved TIMI flow after Abciximab [49]. The use of ic administered Abciximab becomes more accepted during this period, in spite of the fact that no prospective, randomized trials have been conducted so far. In a case from `clinical Decision Making´ in the Journal of Invasive Cardiology the authors had asked how to manage a 63-year-old female with NSTEMI [50]. Amongst the comments from colleagues especially one is interesting in this context. Kereiakes *et al*. suggest that bolus of Abciximab (0.25 mg/kg) should be administered into the target vessel, if the diagnostic angiogram showed impaired coronary flow (TIMI 1-2). PCI should then be performed after 48 hours. Lee *et al*. describes a case report on a 60-year-old man in whom ic Abciximab (instead of using mechanical thrombectomy) was used successfully to dissolve a thrombus that appeared during PCI [51]. In 2007 Carey and colleagues presented a case in which a STEMI-patient, who underwent PCI, was given ic Abciximab in addition to mechanical thrombectomy (aspiration and transluminal extraction catheter atherectomy) [52]. They found that the combination of strategies in this case was beneficial, as they observed early ST-segment resolution, early CK-MB peak, TIMI flow 3 and no evidence of distal embolization. Thus, several case reports and case series have suggested that ic administration of Abciximab is feasible – but based on these reports so far no definite evidence exists that ic administration should be preferred.

As shown in Table **2** our search for clinical trials comparing ic versus iv use of Abciximab resulted in only 5 studies, one small randomised prospective study with one month follow-up [53], one prospective control group matched study with in hospital follow-up [54], one observational study with a partly comparison of iv treated patients from other studies [55] and finally 2 retrospective studies [56,57].

Bellandi *et al*. have presented the only randomized prospective study [53]. In 2004 this study was published in which 45 consecutive patients with STEMI who underwent primary PCI were randomized to either ic or iv treatment with Abciximab. 22 of those were assigned to the ic-group. Angiographic myocardial blush grade, corrected TIMI frame count (CTFC) and reduction in ST segment elevation were used as markers of myocardial reperfusion. To assess initial perfusion defect, final infarct size, myocardial salvage, salvage index, recovery of left ventricle (LV) and myocardial perfusion scintigraphy were performed at admission, after 7 days and 1 month. A significant higher degree of myocardial salvage (ic: 20.4% of LV *vs*. iv: 11.0% of LV; P=0.0001) resulted in higher salvage index in the ic group (0.66 *vs*. 0.40; P=0.003). Also LV recovery improved more in the ic group after 1 month (14.7% *vs*. 8,0%, P=0.013). In addition a decrease in CTFC was observed.

The scintigraphic results led the authors to conclude that administering Abciximab ic in STEMI patients results in an increase of myocardial salvage and better left ventricular recovery.

The latest prospective study on the issue was performed by Ramagnoli *et al*. [54] and published in 2005. 37 consecutive patients with ACS who underwent urgent PCI were given ic Abciximab. This population was matched by baseline characteristics with 37 patients who were treated with iv Abciximab bolus. All 74 patients were routinely pre-treated with iv Abciximab. The 37 patients in the ic group were selected at the discretion of the operator. No differences in angiographic baseline data were found. The angiograms were evaluated independently and blinded by two expert interventional cardiologists with respect to CTFC. In addition cardiac enzymes were collected. Significant decrease in CTFC was observed in the culprit vessel compared to the non-culprit vessel within the ic group in all subgroups (STEMI *vs*. NSTEACS, visible *vs*. no visible thrombus, and TIMI flow 0-1 *vs*. 2-3). The most pronounced improvement was seen when comparing visible *vs*. no visible thrombus with a 37% *vs*. 4% (P=0.008) reduction in CTFC, and TIMI 0-1 versus 2-3 with a 34% *vs*. 21% (P=0.008) reduction in CTFC. A comparison between ic and iv administration of Abciximab was also performed. Baseline CTFC were similar in the ic and iv group. CTFC decreased significantly after ic-administration of Abciximab compared to iv-administration (P=0.001), but only a trend towards improvement was seen in the ic group at the final CTFC (P=0.07). In this comparison cardiac enzymes (CK, CK-MB and TNT) were analysed also. A trend towards reduction of post-treatment peak-values was seen in the ic-group, but only CK-MB in the STEMI-subgroup were found to be significantly different (P=0.03). The authors concluded that CTFC decreases when Abciximab is used ic compared to iv. No clinical follow-up data after discharge were presented.

In the latest study [55], 633 STEMI patients were given ic Abciximab bolus during PCI. The patients were then divided into two subgroups depending on their co-morbidity. Patients with high co-morbidity (failed thrombolysis, cardiopulmonary resuscitation, cardiogenic shock, advanced age or renal failure) were assigned to group II, whereas patients without these risk-factors were assigned to group I. The latter group was compared to earlier studies that compared iv bolus Abciximab to no Abciximab. The authors found that Major Adverse Cardiovascular Events (MACE) occurred in 3.6% in group I which was lower compared to other randomized clinical studies evaluating iv bolus of Abciximab (RAPPORT 5.8% [58], ISAR-2 5.0% [59], ADMIRAL 6.0% [60], CADILLAC 4.4% [61] and ACE 4.5% [62]). A comparison between group I and II was performed with respect to MACE at 30 days and bleeding events. Not surprisingly group II had a higher incidence of both (MACE 3.6% in group I versus 31.9% in group II; bleeding, both minor and major 7.1% in group I versus 15.9% in group II). In conclusion the authors stated that ic use of Abciximab is safe and that the incidence of MACE is lower compared to iv use. The limitation of this study is the observational design, the short follow-up of 30 days and the fact that the patients are compared to studies that were designed to compare Abciximab and placebo. However, the findings support the thesis of ic Abciximab being superior to iv.

In 2004 the study with the longest follow-up was performed. Kakkar *et al*. [56] included 173 patients who had coronary stenting performed and received Abciximab. The population consisted of patients with stable angina, NSTACS or STEMI. At the discretion of the operator, 101 of those received ic bolus of Abciximab, 72 received iv bolus. All subsequently received a 12 h iv infusion of Abciximab. Data were collected retrospectively, and the ic and iv groups were compared. Patient and procedural characteristics in the ic and iv group were similar. Clinical end-points were defined as MI, death, TVR and rehospitalization within 6 months. When comparing the composite end-point of MI and death a borderline significant difference between ic and iv group was found (6% *vs*. 14%, P=0.08). After performing multiple logistic regression controlling for sex, race, diabetes, left ventricular ejection fraction, age, smoking, hypertension and hyperlipidemia, a significant difference was found (ic: 6% *vs*. iv: 14%, P=0.04). No differences were found between the two groups regarding the individual end-points. No safety issues were recorded. The authors noted that ic Abciximab may be superior to iv with respect to the composite end-point of MI and death. However, the conclusion is limited by the fact that the study was relatively small and non-randomised, and that data were collected retrospectively.

The first study comparing ic *vs*. iv use of Abciximab was performed by Wöhrle *et al*. and published in 2003 [57]. In this rather large study of 433 patients with unstable angina, NSTEACS or STEMI, 294 patients had Abciximab bolus administered ic and the remaining 139 patients were treated with iv. The study was retrospective and non-randomized. Baseline characteristics were similar. MACE was defined as death, MI and urgent revascularization within 30 days. The main finding in this study was the significantly lower incidence of MACE (ic: 10% *vs*. iv: 20%, P=0.008) which was probably part of the motivation for the later studies described above.

## FUTURE STUDIES

The studies described above all have in common that the authors call for randomized prospective studies to evaluate the efficacy and safety of ic administration of Abciximab. By searching www.clinicaltrials.gov we only found one ongoing study [63].

The hypothesis in this study is that ic bolus Abciximab given during primary PCI in a STEMI population leads to higher iv concentration and thus improved epicardial flow and perfusion, and reduction of no-reflow and infarct size, and subsequently better outcome compared to iv bolus. The study is no longer recruiting participants.

At our centre at Gentofte University Hospital, Copenhagen, we are conducting a study with the purpose to evaluate the efficacy and safety of ic bolus Abciximab compared to iv bolus. To do so we plan to include 500 patients (of which 360 have already been included) in a prospective randomised trial. Patients scheduled for PCI and who meet the usual criterias for Abciximab will be randomised to either ic or iv bolus Abciximab. All patients receive the standard 12 h post-PCI Abciximab iv infusion. Follow-up is after 1 month and one year. Clinical end-points are death, MI, angina, TVR and stroke. Incidence of bleeding is recorded. For further information visit www.clinicaltrials.gov NCT00685464.

## CONCLUSION

Whether the optimal route of bolus Abciximab is iv or ic is still unclear. Existing evidence arguing for ic Abciximab is based first on relevant pharmacological rationale, second on a number of case reports, and third on 5 clinical studies on which no final conclusion can be drawn.

Therefore, randomised prospective studies testing the relevant hypothesis, that ic administration may be superior, are needed.

## Figures and Tables

**Table 1:** Mechanisms of Abciximab

IIb/IIIa platelet glycoprotein blockade determining platelet aggregation inhibition; active thrombolytic effect by means of partial displacement of platelet-bound fibrinogen; inhibition of platelet-induced generation of thrombin reducing granule release: it results in reduced levels of platelet-derived inhibition of fibrinolysis such as PAI-1 and α2-anti-plasmin; Blokade of the binding of the factor XIIIa to platelets, thereby diminishing crosslinking of both fibrin strands and α2-anti-plasmin to fibrin and reduction of clot retraction; blockade of the activated Mac-1 on monocytes and Mac-1-expressing THP-1 cells; inhibition of fibrinogen binding to Mac-1 preventing formation of leukocyte/leukocyte or leukocyte/platelet aggregates; inhibition of Mac-1-mediated monocyte adhesion on ICAM-1; inhibition of factor X binding to Mac-1 and Mac-1-mediated conversion of factor X to Xa; blockade of αVb3 vitronectin receptor preventing smooth muscle cells migration and intimal hyperplasia following vascular injury, inhibition of platelet adhesion to osteopontin in atherosclerotic plaque and platelet-mediated thrombin generation via blokade of αVb3 receptor.

After Romagnoli *et al.* [41].

**Table 2:** Trials Evaluating Intravenous *vs.* Intracoronary Abciximab

Author Year [ref.]	N^°^ of Patients Studied iv/ic	Population	Design	Follow-up	Evaluation	Conclusion
Belandi 2004 [53]	45 23/22	STEMI	Prospective. Randomized.	7 days. 1 month.	IS, MS, SI, left ventricular function recovery after 1 month.	↓ in IS, ↑ in MS, SI and LVEF in ic-group*.
Romagnoli 2005 [54]	74 37/37	STEMI NSTEACS	Prospective. Control-group matched by baseline-characteristics.	In-hospital.	Angiographic by CTFC. Cardiac enzymes (CK, CK-MB, TNT).	↓ in CTFC*. Trend towards ↓ of peak enzyme values in ic-group. ↓ of CK-MB peak value in STEMI-subgroup in ic-group*.
Wöhrle 2007 [55]	633	STEMI	Observational. Control: Other studies with no or iv-Abciximab	30 days.	MACE (death, myocardial infarction, urgent TVR). Bleeding.	↓ MACE in present study vs. earlier iv-studies. No safety issues.
Kakkar 2004 [56]	173 72/101	Stable angina STEMI NSTEACS	Retrospective.	6 months.	MACE (death, myocardial infarction).	MACE ↓ in ic-group, 5.8% vs. 13.9%*.
Wöhrle 2003 [57]	403 109/294	Unstable angina STEMI NSTEACS	Retrospective.	30 days.	MACE (death, myocardial infarction, urgent revascularization).	MACE ↓ in ic-group, 10.2% vs. 20.2%*.

* P<0.05.

Abbreviations: CK: Creatin-kinase; CK-MB: Creatin-kinase-MB; CTFC: Corrected TIMI frame count; IS: Infarct Size; MACE: Major Adverse Cardiovascular events; MS: Myocardial Salvage; NSTEACS: non-ST-elevation acute coronary syndrome; SI: Salvage Index; STEMI: ST-elevation myocardial infarction; TNT: Troponin T; TVR: Target vessel revascularization.
